# Crystal structure of the putative cyclase IdmH from the indanomycin nonribosomal peptide synthase/polyketide synthase

**DOI:** 10.1107/S2052252519012399

**Published:** 2019-10-24

**Authors:** Ieva Drulyte, Jana Obajdin, Chi H. Trinh, Arnout P. Kalverda, Marc W. van der Kamp, Glyn R. Hemsworth, Alan Berry

**Affiliations:** a Astbury Centre for Structural Molecular Biology and School of Molecular and Cellular Biology, University of Leeds, Leeds LS2 9JT, England; b School of Biochemistry, University of Bristol, University Walk, Bristol BS8 1TD, England

**Keywords:** polyketide biosynthesis, antibiotics, *Steptomyces antibioticus*, cycloaddition, crystal structure, protein crystallography, QM/MM reaction modelling, Diels–Alder reaction

## Abstract

Indanomycin is biosynthesized by a hybrid nonribosomal peptide synthase/polyketide synthase (NRPS/PKS) followed by a number of ‘tailoring’ steps to form the two ring systems that are present in the mature product. It had previously been hypothesized that the indane ring of indanomycin was formed by the action of IdmH using a Diels–Alder reaction. Here, the crystal structure of a selenomethionine-labelled truncated form of IdmH (IdmH-Δ99–107) was solved using single-wavelength anomalous dispersion (SAD) phasing. This truncated variant allows consistent and easy crystallization, but importantly the structure was used as a search model in molecular replacement, allowing the full-length IdmH structure to be determined to 2.7 Å resolution. IdmH is a homodimer, with the individual protomers consisting of an α+β barrel. Each protomer contains a deep hydrophobic pocket which is proposed to constitute the active site of the enzyme. To investigate the reaction catalysed by IdmH, 88% of the backbone NMR resonances were assigned, and using chemical shift perturbation of [^15^N]-labelled IdmH it was demonstrated that indanomycin binds in the active-site pocket. Finally, combined quantum mechanical/molecular mechanical (QM/MM) modelling of the IdmH reaction shows that the active site of the enzyme provides an appropriate environment to promote indane-ring formation, supporting the assignment of IdmH as the key Diels–Alderase catalysing the final step in the biosynthesis of indanomycin through a similar mechanism to other recently characterized Diels–Alderases involved in polyketide-tailoring reactions. An animated Interactive 3D Complement (I3DC) is available in Proteopedia at https://proteopedia.org/w/Journal:IUCrJ:S2052252519012399.

## Abstract 2

The crystal structure of IdmH from the biosynthetic gene cluster for indanomycin is presented. NMR data show that this enzyme binds its postulated product, indanomycin, and QM/MM modelling of the reaction strongly supports the view that IdmH catalyses indane-ring formation in indanomycin biosynthesis via a Diels–Alder reaction.

## 1. Introduction   

Polyketides and nonribosomal peptides are two major classes of natural products which give rise to nearly one third of the current pharmacopoeia (Newman & Cragg, 2012 ▸). They exhibit high structural diversity and are proven to be excellent therapeutics, but there is an increasing interest in diversifying current natural product libraries to produce analogues with improved or novel biological activity (Cummings *et al.*, 2014 ▸). Natural products classed as polyketides, nonribosomal peptides and hybrids of both are often biosynthesized by giant, complex enzymes known as polyketide synthases (PKS) and nonribosomal peptide synthetases (NRPS). Both systems consist of a range of domains which incorporate a number of starter and extender units to build a linear structure. In the case of polyketides, the linear enzyme products can then be further tailored by various enzymes, including cyclases, oxidases, reductases and methylases, to yield a diverse array of bioactive compounds (Olano *et al.*, 2010 ▸).

**Figure 1:**
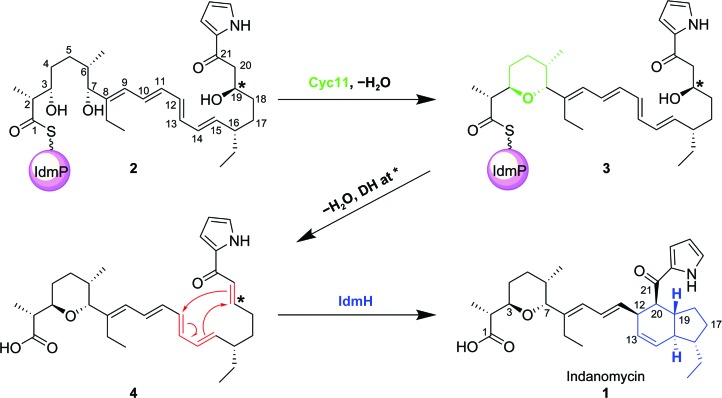
The proposed mechanism for indanomycin maturation through the formation of tetrahydropyran (green) and indane (blue) rings. After ‘starter’ pyrrole biosynthesis, the polyketide is built through the actions of the five Idm ORFs IdmL–P, and the final polyketide chain is left attached to IdmP. Cyc11 is thought to mediate the formation of the tetrahydropyran ring through a direct nucleophilic replacement to generate **3** (Li *et al.*, 2009 ▸). This reaction could then be followed by hydrolysis to release **4** from the IdmP subunit. Finally, indane-ring formation is thought to be mediated by a putative cyclase, IdmH (Li *et al.*, 2009 ▸). For [4+2] cycloaddition to occur, C19 of **2** (denoted with an asterisk) needs to be dehydrated to produce a double bond to act as the dienophile (Rommel *et al.*, 2011 ▸). Since the second PKS module does not contain a dehydratase domain, a hypothetical dehydration step was included between intermediates **3** and **4**. Adapted from Li *et al.* (2009 ▸).

Indanomycin (**1**) is an antibiotic from the pyrroloketo­indane family which is known to act as an effective ionophoric agent against Gram-positive bacteria (Dutton *et al.*, 1995 ▸). Indanomycin is synthesized in *Streptomyces antibioticus* NRRL 8167 by a hybrid nonribosomal peptide synthetase/polyketide synthase (NRPS/PKS; Li *et al.*, 2009 ▸). The NRPS portion of the pathway is proposed to generate a pyrrole moiety from l-proline, which is then extended by the sequential addition of malonyl-CoA, methylmalonyl-CoA and ethylmalonyl-CoA building blocks by ten predicted PKS modules to yield the linear nonribosomal peptide–polyketide hybrid natural product **2** (Fig. 1 ▸). At least two cyclization reactions are then needed to generate the tetrahydropyran and tetrahydroindane rings of indanomycin (**1**; Fig. 1 ▸). The former could be installed while the polyketide is still tethered to the PKS by the cyclase domain, Cyc11, in the terminal, 11th PKS module. Indane-ring formation has been postulated to be catalysed by a separate indane cyclase, IdmH (Li *et al.*, 2009 ▸; Rommel *et al.*, 2011 ▸), using a Diels–Alder [4+2] intramolecular cyclization (Fig. 1 ▸). The feasibility of indane-ring formation in indanomycin proceeding via a Diels–Alder reaction was first shown in its complete chemical synthesis (Edwards *et al.*, 1984 ▸), but to date it has not been possible to characterize the activity of IdmH in molecular detail.

The proposed biosynthetic cycloaddition would require the appropriate diene and dienophile within the intermediate. The indanomycin PKS harbours catalytic domains which would produce the suitable diene intermediate for the cycloaddition to be feasible. However, the required dienophile would not be generated by straightforward use of the catalytic domains present in the indanomycin PKS/NRPS, since the second PKS module is predicted to install a hydroxyl group at C19 (Fig. 1 ▸, compound **2**; Rommel *et al.*, 2011 ▸). To form the appropriate dienophile, dehydration of the alcohol at C19 is therefore needed, either while the linear polyketide is tethered to the synthase or after it has been released. One suggested possibility is that a dehydratase (DH) domain, for example DH3, from a neighbouring module might catalyse the required dehydration in a manner similar to that used in epothilone biosynthesis (Tang *et al.*, 2004 ▸; Li *et al.*, 2009 ▸).

Irrespective of the activity required to dehydrate C19, IdmH has been proposed to catalyse the Diels–Alder reaction which would lead to the final indane-ring formation in indanomycin. In support of this idea, an *idmH* deletion mutant of *S. antibioticus* exhibited both a significant reduction in indanomycin yield and the production of a previously unobserved linear tetraene alternative product (Rommel *et al.*, 2011 ▸). Notably, indanomycin-production levels were restored when the *idmH* gene was introduced to *S. antibioticus* in trans (Rommel *et al.*, 2011 ▸). IdmH is thus thought to be the key cyclase responsible for the formation of the indane ring and hence mature indanomycin. IdmH is a protein consisting of 145 amino acids (Supplementary Fig. S1) exhibiting sequence similarity to a number of natural product-modifying enzymes. These include the epoxide hydroxylase MonBI from *S. cinnamonensis* (50% identity over 14% of the sequence; Minami *et al.*, 2014 ▸) and the polyketide cyclase SnoaL from *S. nogalater* (27% identity over 91% of the sequence; Sultana *et al.*, 2004 ▸) (Supplementary Fig. S2). Both homologues exhibit an α+β barrel fold and are involved in ring closure and aromatic ring hydroxylations in the monensin and nogalamycin biosynthetic pathways, respectively. Beyond polyketide-modifying enzymes, other α+β barrel proteins include Δ^5^-3-ketosteroid isomerase and scylatone dehydratase (Ha *et al.*, 2001 ▸; Lundqvist *et al.*, 1994 ▸), showing that nature has made broad use of this fold in many biosynthetic pathways. Given the diversity of reactions catalysed by enzymes displaying this fold, it is challenging to predict the function of IdmH based on sequence similarity alone.

As a step towards elucidating the underlying enzymatic mechanism of indanomycin maturation, we set out to determine the three-dimensional structure of IdmH. Here, we present crystal structures of IdmH and a deletion variant, IdmH-Δ99–107, at 2.7 and 2 Å resolution, respectively. Structural analysis shows that IdmH has a similar fold to that of the putative hydroxylases SnoAL2 and AcIR (Beinker *et al.*, 2006 ▸) and the polyketide cyclases SnoaL (Sultana *et al.*, 2004 ▸) and AknH (Kallio *et al.*, 2006 ▸). Apparent differences in the putative active site, however, suggest that this enzyme catalyses a distinct reaction from that of its closest structural homologues. We therefore utilized NMR spectroscopy and *in silico* modelling [including quantum mechanical/molecular mechanical (QM/MM) reaction simulations] to probe product binding and the likely reaction catalysed by IdmH, with our analyses supporting the notion that this enzyme is indeed a Diels–Alderase.

## 2. Materials and methods   

### 2.1. Plasmid construction   

The gene sequence encoding the full-length *idmH* gene (ACN69984.1) was amplified by PCR using the genomic DNA from *S. antibioticus* (strain NRRL 8167) as a template, and the forward and reverse primers 5′-ctg gtg ccg cgc ggc agc cat ATG GCT CAT CAG CCT TCG-3′ and 5′-tcc acc agt cat gct agc caT CAC AGG GAC GCC TTC AC-3′, respectively. The amplified sequence contained overhang sequences (in lower case letters) which facilitated ligation to an NdeI-linearized pET-28(a)+ vector using the NEBuilder HiFi DNA-assembly kit (NEB). The generated plasmid also encoded an N-terminal hexa­histidine tag on the protein and a thrombin cleavage site. The gene insert was confirmed by sequencing with T7 and T7term universal primers before transformation into *Escherichia coli* strain BL21(DE3) cells for overexpression. Site-directed mutagenesis to generate the deletion variant IdmH-Δ99–107 was performed using wild-type pET-28_IdmH as a template and the forward and reverse primers 5′-CGC GAC CGC GAG GGG TGG-3′ and 5′-GGA GTG CGT TCC CCG TGC-3′, respectively. The site-directed mutagenesis PCR product was treated with KLD Enzyme Mix (NEB) before transformation into *E. coli* strain 5-α competent cells.

### 2.2. Expression and purification of IdmH   

Recombinant IdmH was expressed in *E. coli* strain BL21(DE3) cells harbouring the pET-28_IdmH plasmid. The cells were grown in 2TY medium containing 50 µg ml^−1^ kanamycin at 37°C to an OD_600_ of 0.4 before lowering the temperature to 15°C. Expression was then induced with 0.4 m*M* isopropyl β-d-1-thiogalactopyranoside (IPTG) at an OD_600_ of 1.0 and the cells were allowed to grow overnight. The cells were harvested by centrifugation at 6000*g* and subsequently resuspended in buffer *A* (50 m*M* Tris–HCl pH 7.4, 0.5 *M* NaCl) containing 20 m*M* imidazole, 0.1 mg ml^−1^ lysozyme, 0.05 mg ml^−1^ DNase and 2 m*M* MgCl_2_. After disrupting the cells with a high-pressure press (Constant Systems), the cell debris was removed by centrifugation at 50 000*g*. The supernatant was then loaded onto a 5 ml HisTrap HP column (GE Healthcare, USA) which had been pre-equilibrated with buffer *A*. Following sample loading, the column was washed with ten volumes of buffer *A* before the target protein was eluted from the column with buffer *A* supplemented with 200 m*M* imidazole. The eluted recombinant protein was desalted into buffer *B* (10 m*M* Tris–HCl pH 7.2, 50 m*M* NaCl) using a PD-10 desalting column (GE Healthcare, USA) and the hexahistidine tag was then removed by incubating the sample with 1 U thrombin (GE Healthcare, USA) per milligram of recombinant IdmH overnight at 4°C. Thrombin was removed by loading the protein mixture onto a 5 ml HiTrap Q column (GE Healthcare, USA) equilibrated with buffer *B*. The column was washed with five column volumes of buffer *B* before a linear gradient of increasing NaCl concentration was applied to elute the bound proteins. 2.5 ml fractions were collected across the gradient, which were analysed by SDS–PAGE. Fractions containing the protein were pooled, concentrated and applied onto a size-exclusion chromatography column (HiLoad 26/600 Superdex 75 pg, GE Healthcare, USA) pre-equilibrated with buffer *B*. After a void volume of 40 ml, 1 ml fractions were collected. These were once more analysed using SDS–PAGE and the fractions containing the most highly pure IdmH were combined to form the final sample. This was concentrated to 20 mg ml^−1^ (as determined from the *A*_280_ with an extinction coefficient of 17 990 *M* ^−1^ cm^−1^) using a centrifugal filter concentration device (Vivaspin 20; 10 000 molecular-weight cutoff). Finally, samples were flash-frozen in liquid nitrogen and stored at −80°C until use. The IdmH-Δ99–107 variant was expressed and purified in exactly the same way.

Production of selenomethionine-labelled IdmH-Δ99–107 was achieved by cultivation of the methionine-auxotrophic *E. coli* strain B834 (DE3) cells in M9 minimal medium supplemented with BME vitamins (Sigma–Aldrich) and 50 µg ml^−1^ l-(+)-selenomethionine (Anatrace). All expression and purification steps were performed as for the native protein. Incorporation of selenomethionine was confirmed by LC/MS analysis.

### 2.3. Protein crystallization, X-ray data collection and structure determination   

Initial crystallization conditions for full-length IdmH were identified with The JCSG Core Suites I–IV (Qiagen) using Formulatrix NT8 robotic mixing of protein at 20 mg ml^−1^ with crystallization solution at 1:1, 2:1 and 1:2 volumetric ratios in separate drops. IdmH crystal hit conditions were subsequently optimized with respect to pH and precipitant concentration using hanging-drop vapour diffusion, giving the crystals used for data collection in 0.2 *M* calcium acetate, 0.1 *M* MES pH 5.5, 18.6%(*w*/*v*) polyethylene glycol 8000. The crystals were cryoprotected by soaking them in mother-liquor solution containing 20%(*v*/*v*) glycerol for about 30 s before flash-cooling them by plunging them into liquid nitrogen. X-ray diffraction data for full-length IdmH were collected on beamline I04 at Diamond Light Source.

The structure of IdmH could not be determined by molecular replacement using these data and the structure was therefore solved using selenomethionine-labelled IdmH-Δ99–107. Crystals were obtained in 3.6–4 *M* sodium formate, 10–15%(*v*/*v*) glycerol using the same approach as described for the wild-type enzyme. The crystals were flash-cooled in liquid nitrogen for data collection, this time in the absence of cryoprotectant. X-ray diffraction data for selenomethionine-labelled IdmH-Δ99–107 were recorded on MASSIF-1 at the European Synchrotron Radiation Facility (Bowler *et al.*, 2015 ▸, 2016 ▸; Svensson *et al.*, 2015 ▸, 2018 ▸; Nurizzo *et al.*, 2016 ▸), while native IdmH-Δ99–107 data were recorded on beamline I03 at Diamond Light Source.

All diffraction data were indexed and integrated using *XDS* (Kabsch, 2010 ▸) before subsequent scaling in *AIMLESS* and data processing using the *CCP*4*i*2 graphical user interface to *CCP*4 (Potterton *et al.*, 2018 ▸). The structure of IdmH-Δ99–107 was determined to 2 Å resolution by single-wavelength anomalous dispersion (SAD) phasing using the *SHELX* pipeline followed by density modification using *Parrot* and automated model building in *Buccaneer*. Iterative rounds of manual model building and refinement were then performed in *Coot* and *REFMAC*5, respectively (Cowtan, 2006 ▸, 2010 ▸, 2012 ▸; Murshudov *et al.*, 2011 ▸; Sheldrick, 2010 ▸). The structure of wild-type IdmH was solved by molecular replacement using a single monomer of IdmH-Δ99–107 as a search model in *Phaser* (McCoy, 2007 ▸; McCoy *et al.*, 2007 ▸). The final model was once more generated following iterative rounds of manual model building in *Coot* and refinement using *REFMAC*5 imposing local noncrystallographic symmetry (NCS) restraints throughout (Emsley & Cowtan, 2010 ▸; Murshudov *et al.*, 2011 ▸). The model was validated using *MolProbity* (Chen *et al.*, 2010 ▸). All data-collection and structure-refinement statistics are listed in Table 1 ▸.

### 2.4. NMR spectroscopy   

950 MHz Bruker Ascend Aeon and 750 MHz Oxford NMR spectrometers equipped with cryogenically cooled triple-resonance probes were used for all NMR experiments. Spectra were recorded at 25°C in NMR buffer [20 m*M* Tris pH 7.3, 50 m*M* NaCl, 1 m*M* DTT, 0.02%(*w*/*v*) NaN_3_, 5%(*v*/*v*) D_2_O]. 2D NMR experiments were carried out using Wilmad NMR tubes (Precision, limit 600 MHz frequency), while Shigemi NMR tubes were used for triple-resonance experiments.

#### 2.4.1. Backbone nuclei resonance assignment   

Triple-labelled protein was obtained by growing *E. coli* BL21(DE3) cells containing the wild-type IdmH expression plasmid in M9 medium prepared in D_2_O and supplemented with [^13^C]-d-glucose and ^15^NH_4_Cl (Cambridge Isotope Laboratories). Labelled protein was purified as described for the wild type, except that no thrombin-cleavage step was carried out. A set of 3D BEST-TROSY backbone resonance assignment spectra [HNCA, HN(CO)CA, HNCO, HN(CA)CB and HN(CO)CACB] were recorded at 750 MHz, while a 3D BEST-TROSY HN(CA)CO spectrum was recorded at 950 MHz, using pulse sequences (Solyom *et al.*, 2013 ▸) obtained from the Institut de Biologie Structurale (IBS), Grenoble, France for [^15^N,^13^C,^2^H]-labelled IdmH at a concentration of 0.8 m*M*. Non-uniform sampling was used to speed up the acquisition time and obtain high-resolution spectra.

The raw spectra were pre-processed with *NMRPipe* and *NMRDraw* (Delaglio *et al.*, 1995 ▸). Non-uniform sampled data were reconstructed using the *MddNMR* suite (Jaravine & Orekhov, 2006 ▸; Jaravine *et al.*, 2008 ▸). Backbone nuclei resonance assignment was performed using *CCPNmr Analysis* (Vranken *et al.*, 2005 ▸) and *AutoAssign* (Zimmerman *et al.*, 1997 ▸).

#### 2.4.2. Chemical shift perturbation studies   

^15^N-labelled protein was prepared by growing *E. coli* strain BL21(DE3) containing the wild-type *idmH* expression plasmid in M9 minimal medium using ^15^NH_4_Cl as the sole nitrogen source. ^1^H–^15^N HSQC-TROSY spectra were collected at an IdmH concentration of 0.2 m*M* with varying concentrations of indanomycin (0–200 µ*M*) at 25°C at a field strength of 750 MHz. The raw data were pre-processed using *TopSpin* (Bruker). The NMR spectra were analysed using *CCPNmr Analysis* (Vranken *et al.*, 2005 ▸). The minimal chemical shift perturbation was calculated from ^1^H and ^15^N chemical shift differences using 0.154 as a scaling factor for ^15^N shift changes and calculating the closest chemical shift distance between peaks in the spectra with and without indanomycin (Williamson, 2013 ▸).

### 2.5. *In silico* modelling   

Molecular docking of indanomycin (**1**) and an analogue truncated at C8 was performed using *AutoDock Vina* 1.1.2 (Trott & Olson, 2010 ▸). *AutoDockTools* 1.5.4 was used to merge the nonpolar hydrogens and define all formally single bonds as rotatable bonds for docking. Subsequently, docking was performed with *AutoDock Vina* using a grid of 25 × 20 × 25 Å centred on the active-site cavity (in either chain *A* or chain *B* of the crystal structure). Three top-scoring poses in different orientations were selected by filtering out highly similar poses and poses that did not place the indane ring inside the cavity. Subsequently, the protein–product poses (with the complete indanomycin) were prepared for molecular-dynamics simulation with *AmberTools*17 (Case *et al.*, 2017 ▸) using the *Enlighten* protocols (see https://github.com/marcvanderkamp/enlighten). For one of the poses, a different hydrogen-bonding network was obtained by ‘flipping’ the Asn117 side chain. Simulations were thus performed on four different initial protein–product complexes. The *PREP* protocol was used to prepare the protein–ligand system for simulation, which involved (i) adding hydrogens [all residues in their standard protonation states in line with prediction by *PROPKA* 3.1 (Olsson *et al.*, 2011 ▸; Søndergaard *et al.*, 2011 ▸); His44 singly protonated on H^δ1^ with all other His singly protonated on H^∊2^ as predicted by the *AmberTools* program *reduce*], (ii) parameterization of indanomycin using AM1-BCC partial charges and GAFF parameters assigned by *Antechamber* (Wang *et al.*, 2004 ▸, 2006 ▸) and (iii) solvation of the active site with TIP3P water molecules (in a 20 Å sphere centred on the N atom of indanomycin, in addition to crystallographically determined water molecules). After a brief structural optimization (using the *STRUCT* protocol, which involves simulated annealing and energy minimization), a molecular-dynamics simulation of 200 or 300 ps was performed for each pose (using the *DYNAM* protocol at a constant temperature of 298 K).

Snapshots from molecular-dynamics simulations between 100 and 300 ps were used to start approximate QM/MM umbrella sampling simulations of the reverse Diels–Alder reaction (at least 10 ps apart) using the same protocol as used previously (Byrne *et al.*, 2016 ▸) using DFTB2 (Elstner *et al.*, 1998 ▸) for indanomycin (QM region) and the Amber force field ff14SB (Maier *et al.*, 2015 ▸) for the enzyme environment. In short, the reaction coordinate used to follow the Diels–Alder reaction was the distance between the centre of mass of the dienophile carbons (C19 and C20) and the centre of mass of the diene carbons to which they bond (C15 and C12). By using this reaction-coordinate definition, no bias is applied as to which carbon–carbon bond is formed or broken first (or indeed whether or not the bonds are formed/broken synchronously). Umbrella sampling was performed from reaction coordinate value 1.2 to value 3.8 Å (in steps of 0.1 Å, with additional windows at 2.15, 2.25 and 2.35 Å). The same conditions were used as in the *DYNAM* protocol, apart from using a 1 fs timestep (instead of 2 fs). Snapshots were saved every 0.5 ps. A restraint of 100 kcal mol^−1^ Å^−1^ and 2 ps of simulation was used for each umbrella sampling window. Reaction-coordinate values were recorded every 1 fs and used for input to the Weighted-Histogram Analysis Method (WHAM; Grossfield, 2013 ▸), resulting in the potential of mean force (free energy) along the reaction coordinate (bin width of 0.05 Å). For each docking pose, 11 QM/MM reaction simulations were performed, each starting from a different snapshot. The QM/MM protocols are designed to limit computational resources and are thus approximate. Our aim is not to analyse the reaction in detail, but to distinguish between different possible reactive binding poses. For example, the use of a single, combined reaction coordinate (obtaining a 1D-PMF) is less accurate than a 2D-PMF with sampling along two reaction coordinates representing the carbon–carbon bonds formed [see, for example, Świderek & Moliner (2016 ▸), where this is discussed in detail]. With the combined reaction coordinate used here, reasonable sampling is obtained along the minimum free-energy region indicated in a 2D-PMF (see Supplementary Fig. S3). Although longer sampling (for example 20 ps per window after a 2 ps equilibration) affects the free-energy barriers for simulations, we have tested that this does not significantly change the differences in the barrier between different poses (similar to as reported by Hirvonen *et al.*, 2019 ▸). To obtain representative snapshots for the approximate transition state, the centroid of the highest populated cluster was used, as obtained with hierarchical agglomerative clustering on the r.m.s.d. of the indanomycin atoms after alignment on the protein backbone on the structures sampled in the windows with reaction-coordinate restraints at 2.2 and 2.3 Å.

## 3. Results   

### 3.1. Structural determination of IdmH-Δ99–107 and native IdmH   

The N-terminally hexahistidine-tagged wild-type IdmH was successfully expressed in *E. coli* strain BL21(DE3) cells and purified to homogeneity, and the hexahistidine tag was removed by thrombin cleavage. Size-exclusion chromatography and LC/MS analysis (Supplementary Fig. S4) suggested a dimeric oligomeric state for this protein. Initial attempts at crystallization yielded small needle-like crystals, but optimization of these initial conditions produced larger rod-shaped crystals. X-ray diffraction data were collected to 2.7 Å resolution, but attempts to determine the structure by molecular replacement were unsuccessful, presumably owing to the lack of a suitable search model and the large number of molecules in the asymmetric unit as estimated using *MATTPROB* (Kantardjieff & Rupp, 2003 ▸).

**Figure 2:**
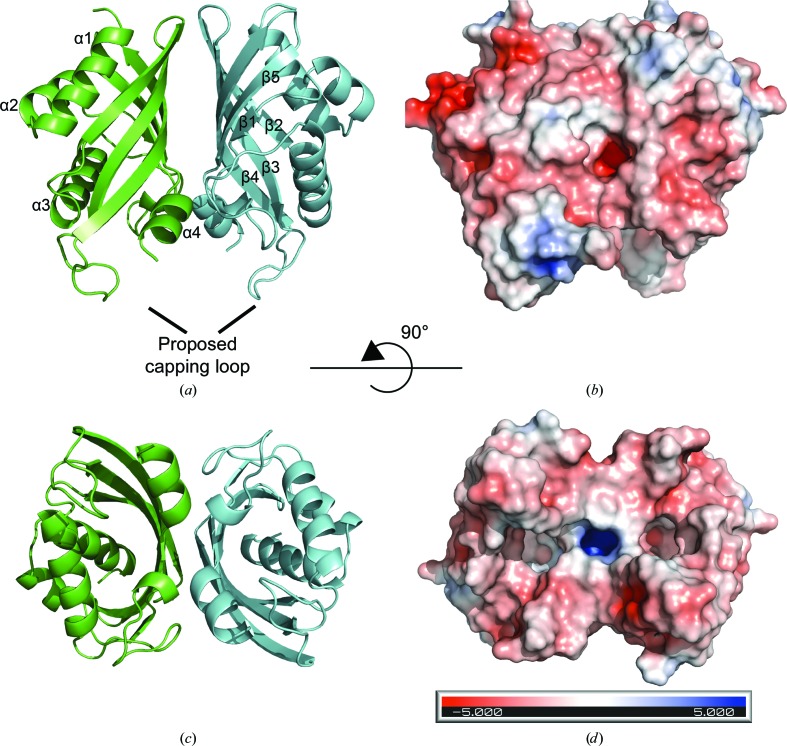
The structure of IdmH. (*a*, *c*) Ribbon diagrams showing the IdmH dimer architecture. Individual monomers are coloured green and blue, respectively. The overall shape of the monomer can be described as a distorted α/β-barrel with the dimerization interface located between the β-sheets. Each monomer comprises four α-helices (α1–α4) and five β-strands (β1–β5). (*b*, *d*) Surface representation of the IdmH dimer. The surface is coloured using *APBS* (Jurrus *et al.*, 2018 ▸) according to the approximate electrostatic potential from −5 *kT* e^−1^ (red) to 5 *kT* e^−1^ (blue).

To improve crystallization, a number of variants were produced to reduce the surface entropy (Goldschmidt *et al.*, 2007 ▸) or to truncate predicted surface loops (Roy *et al.*, 2010 ▸; Supplementary Fig. S1 and Table S1). One variant, containing a deletion of residues 99–107, resulted in large, easily reproducible crystals in the cubic space group *F*23 [Supplementary Fig. S5(*b*)] from new crystallization conditions. This protein was also produced labelled with selenomethionine and crystallized under identical conditions, allowing the crystal structure of IdmH-Δ99–107 to be determined to 2 Å resolution using selenium SAD phasing. The asymmetric unit comprises one copy of the IdmH-Δ99–107 dimer with the entire polypeptide chain successfully modelled into density, except for the N- and C-terminal regions. Both the selenomethionine-labelled protein structure and the isomorphous native structure of IdmH-Δ99–107 were modelled independently (Table 1 ▸), giving almost identical structures that superpose with an r.m.s.d. of 0.12 Å over 257 amino acids. The remainder of the structural description will focus on the unlabelled native protein as this should best represent the natural form of the enzyme. With this structure in hand, the crystal structure of wild-type IdmH was determined by molecular replacement using a single subunit of IdmH-Δ99–107 as a search model (Fig. 2 ▸). Electron-density maps were well defined for all ten protomers in the asymmetric unit, allowing all chains to be modelled, with the exception of some residues at the extreme N- and C-termini (residues in the ranges 1–6 and 143–148). The final model was refined at 2.7 Å resolution with an *R*_work_ of 0.21 and an *R*_free_ of 0.24. Both models were checked with *MolProbity*, revealing a high-quality model (*MolProbity* scores of 1.00 and 0.91 for wild-type IdmH and IdmH-Δ99–107, respectively) with no Ramachandran outliers.

### 3.2. Analysis of the IdmH structure   

#### 3.2.1. The overall fold   

The ten molecules in the asymmetric unit for the wild-type protein form five almost identical dimers. The following discussion of the IdmH structure will therefore focus on the dimer formed by chains *A* and *B* unless otherwise stated, as this represents the most well ordered dimer based on an analysis of the overall *B* factors of the chains. The structure of IdmH reveals a homodimer, with individual protomers consisting of a core α+β barrel. Each subunit is composed of a five-stranded mixed β-sheet with four strands (β2–β5) continuous in sequence and an additional β-strand (β1), considerably shorter in length, provided later in the polypeptide chain (Fig. 2 ▸). The β-sheet of each subunit is curved and forms the central part of a distorted α+β barrel. The barrel is completed by three α-helices (α1–α3) on the periphery of the dimer and a fourth short helix (α4) completing the fold. The dimer assembly is driven by hydrophobic interactions between the β-sheets from the two sub­units, generating the dimer shown in Fig. 2 ▸.

At the centre of each protomer, a large hydrophobic pocket is found which is proposed to form the IdmH active site (see below). Two loops (residues 38–46 and 99–108) are located near to the entrance of this hydrophobic pocket and limit the access to the proposed active site from the bulk solvent. Interestingly, the loop, which comprises residues 99–108 and is partially truncated in the IdmH-Δ99–107 variant, appears to occupy two distinct conformations within each of the five dimers in the asymmetric unit. Indeed, in each dimer each protomer has this loop occupying either a more open or a more closed conformation above the hydrophobic cavity. Whilst we cannot rule this out as an artefact of the crystallization, this suggests that this loop is likely to be highly dynamic in solution and may also hint at a potential change in conformation between the two protomers, suggesting the potential for allostery in this enzyme (Supplementary Fig. S6). Furthermore, studies of a number of Diels–Alderases have shown that a loop covering the active site becomes more ordered upon substrate binding and this loop could also play such a role here (Byrne *et al.*, 2016 ▸; Zheng *et al.*, 2016 ▸).

#### 3.2.2. The proposed active site   

**Figure 3:**
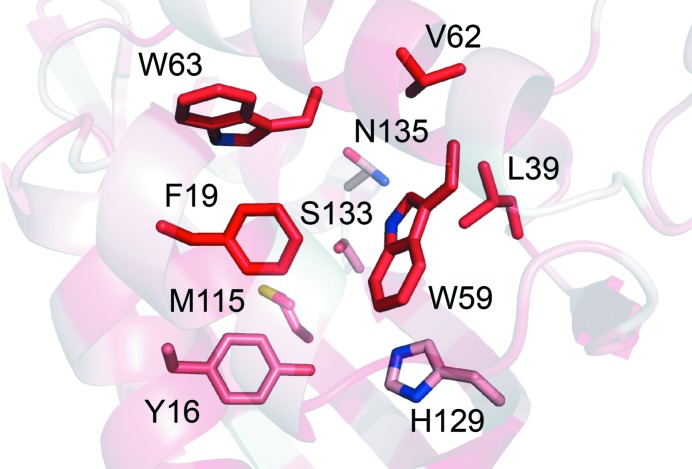
Detailed view of the putative IdmH active site, highlighting key residues. The abundance of aromatic/hydrophobic residues in the active site might play a role in the catalysis of indane-ring formation by guiding the mainly hydrophobic substrate into an appropriate conformation for catalysis. The residues are coloured according to the normalized consensus hydrophobicity scale, where the most hydrophobic residues are red and the most hydrophilic residues are white (Eisenberg *et al.*, 1984 ▸).

A deep hydrophobic pocket penetrating towards the core of the barrel can be observed in each IdmH protomer. It is likely that this constitutes the active site of the enzyme (Fig. 3 ▸ and Supplementary Fig. S7). Each pocket is lined by the side chains of residues Tyr16, Phe19, Leu39, Trp59, Val62, Trp63, Met115, His129, Ser133 and Asn135. We propose that the preponderance of hydrophobic residues might act to steer the hydrophobic substrate (**4** in Fig. 1 ▸) into an appropriate conformation for facile ring cyclization by acting as a natural product template, as observed by others in structurally related cyclases (Barajas *et al.*, 2017 ▸; Li *et al.*, 2010 ▸; Liu *et al.*, 2015 ▸; Carey & Sundberg, 2007 ▸).

#### 3.2.3. Sequence and structural homologues of IdmH   

*BLASTp* (Supplementary Fig. S2) and *DALI* searches against the PDB revealed that the closest sequence and structural homologue to IdmH is the putative polyketide cyclase from *Chromobacterium violaceum* (PDB entry 4lgq; 30% sequence identity; Joint Center for Structural Genomics, unpublished work). Many of the closest structural and sequence matches, however, stem from structural genomics efforts and thus lack accompanying biochemical data and publications supporting their functional annotation. The putative hydrolases AcIR (PDB entry 2gey) and SnoaL2 (PDB entry 2gex) from *S. galilaeus* and *S. nogalater*, respectively (Beinker *et al.*, 2006 ▸) as well as the polyketide cyclase SnoaL (PDB entry 1sjw; Sultana *et al.*, 2004 ▸) did appear as close structural matches to the structure of IdmH even though they share at best 28% sequence identity. We therefore focused our structural comparisons against these biochemically characterized enzymes.

**Figure 4:**
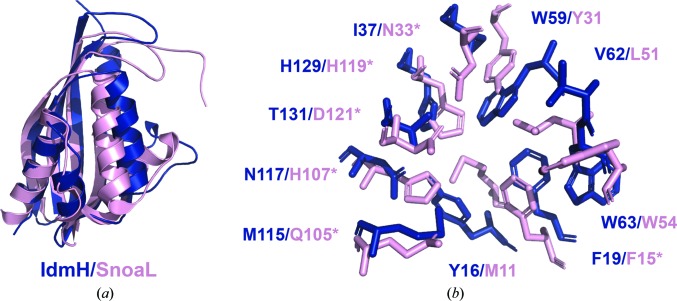
Comparison of IdmH (blue) with its sequence and structural homologue SnoaL (pink). (*a*) IdmH chain *A* was aligned with SnoaL (PDB entry 1sjw) chain *A* using *PyMOL*. (*b*) Active-site residues in SnoaL (pink) compared with their counterparts in IdmH (blue). While some residues appear to be conserved between the two proteins (His129/His119, Trp63/Trp54 and Phe19/Phe19), the charged essential catalytic residues His107 and Asp121 are unique to SnoaL. Another difference between the two active sites arises from different choices and positioning of aromatic residues (Trp59/Tyr31 and Trp63/Trp54), while Tyr16 appears to be completely unique to IdmH. Asterisks denote SnoaL catalytic residues (Sultana *et al.*, 2004 ▸).

We superimposed the structure of IdmH on its sequence homologues using *PyMOL* (Schrödinger) and found that the polyketide cyclase SnoaL from the nogalamycin biosynthetic pathway (PDB entry 1sjw; Sultana *et al.*, 2004 ▸) exhibited the highest structural similarity to IdmH as measured by the root-mean-square deviation of the fit (r.m.s.d. = 1.56 Å over 549 atoms) [Fig. 4 ▸(*a*)]. Crystallographic analysis and gel-filtration chromatography experiments both suggest that SnoaL forms a tetramer in solution (Sultana *et al.*, 2004 ▸), in contrast to IdmH which is homodimeric. SnoaL catalyses an intramolecular aldol condensation using the conserved residue Asp121 as an acid/base catalyst during the reaction. Other essential residues for the activity of SnoaL include Asn33, Gln105 and two histidines, His107 and His119. In contrast, the active site of IdmH does not contain residues that can perform acid/base chemistry at equivalent positions to the catalytic Asp121 and His107 of SnoaL, but rather contains Thr131 and Asn117 instead [Fig. 4 ▸(*b*)]. Of note, Thr131 is implicated as important in the suggested catalytic mechanism of IdmH discussed later. Both active sites include many aromatic residues, and some of them are conserved between the two proteins (for example Phe19 in IdmH and Phe15 in SnoaL and His129 in IdmH and His119 in SnoaL). There are also two tryptophan residues (Trp63 in IdmH and Trp54 in SnoaL) that appear to be conserved between these proteins; however, they appear to be in entirely different orientations. These different orientations could reflect genuine differences between these two structures or they may arise from the fact that the SnoaL structure contains a bound substrate. Furthermore, there are additional tryptophan (Trp59) and tyrosine (Tyr16) residues in IdmH which seem to be unique to this protein (SnoaL contains Tyr31 and Met11 at these locations) [Fig. 4 ▸(*b*)]. Taken together, these data suggest that IdmH is unlikely to catalyze an aldol condensation as found for SnoaL and is likely to interact with its substrate in a different manner.

### 3.3. Investigating IdmH enzymatic activity by nuclear magnetic resonance spectroscopy   

We had no access to the potential IdmH substrate **4**, so we chose to investigate the binding of the product, indanomycin (**1**), to the enzyme by 2D nuclear magnetic resonance (NMR) spectroscopy. Although this did not allow us to directly test the hypothesis that IdmH catalyses indane-ring formation, we postulated that the enzyme would bind the product of the reaction, providing evidence to support its role in indanomycin biosynthesis.

**Figure 5:**
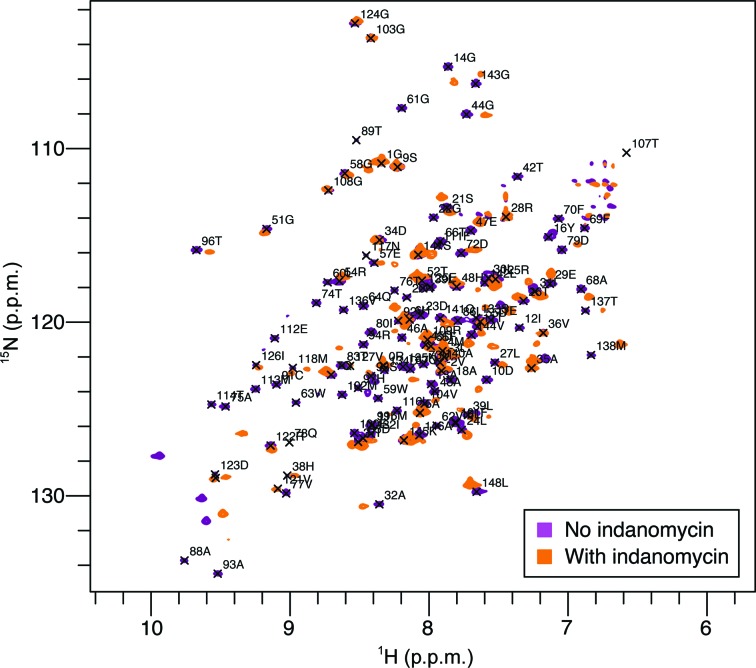
^1^H–^15^N HSQC-TROSY spectra of [^15^N]-labelled IdmH alone (purple) and in the presence of a stoichiometric amount of the ligand indanomycin (orange). Upon the addition of the ligand indanomycin, significant changes in the chemical shifts of some peaks were observed.

IdmH was labelled with ^13^C, ^15^N and ^2^H and, following the collection and processing of a number of triple-resonance spectra (Yamazaki *et al.*, 1994 ▸; Supplementary Table S2), the NMR resonances of backbone nuclei were assigned for 88% of residues. To investigate indanomycin binding, [^15^N]-IdmH was mixed with a stoichiometric amount of ligand (indanomycin) and a ^1^H–^15^N HSQC-TROSY spectrum was acquired (Fig. 5 ▸). Significant chemical shift perturbations (CSPs) for a number of backbone amides compared with the native protein were seen on addition of indanomycin, suggesting a binding event.

**Figure 6:**
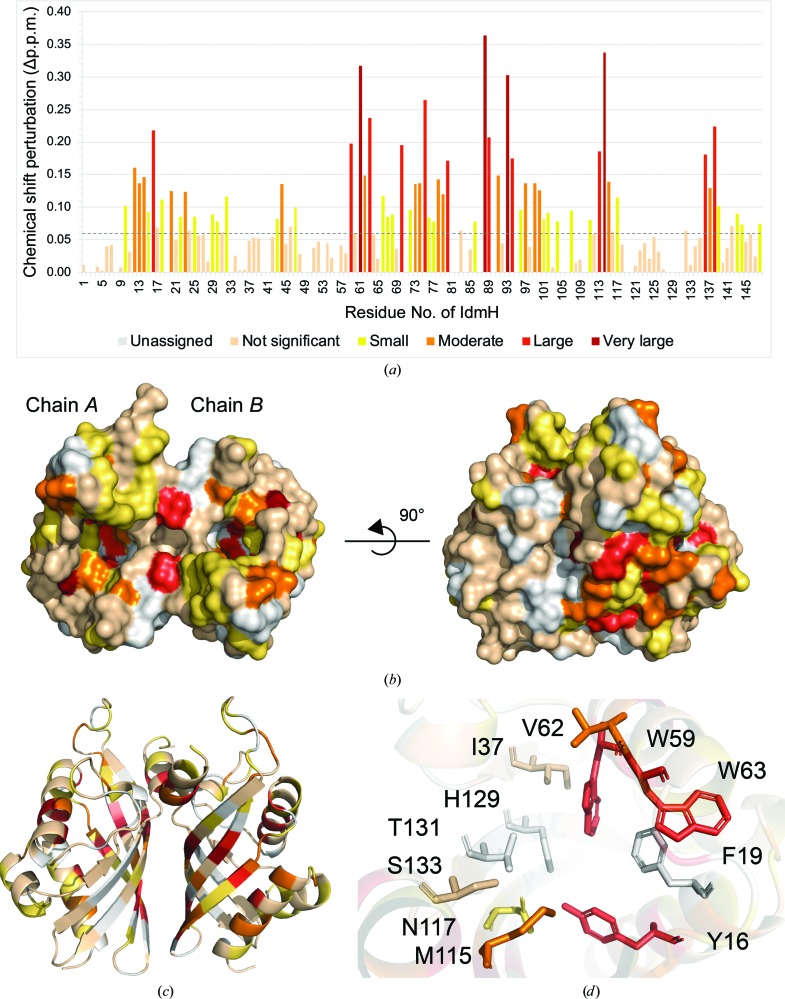
Chemical shift perturbations (CSPs) mapped onto IdmH following the addition of indanomycin. (*a*) Histogram showing the geometrical distance moved by each peak assigned to a residue. Peaks are coloured from yellow to dark red as the value of the CSP increases. Chemical shifts which were smaller than the standard deviation of all shifts (0.072 p.p.m.) are coloured pale cream. Surface (*b*) and ribbon (*c*) representations of IdmH with peak shifts mapped using the same colour scheme as in (*a*). Residues which could not be assigned in the NMR spectrum are coloured white. (*d*) Active-site cavity of chain *B* with the proposed catalytic residues coloured according to the same colour scheme as in (*b*) and (*c*).

To investigate the binding further and to localize the indanomycin-binding site, titration spectra with substoichiometric ligand:protein ratios were recorded. Significant shifts in both the ^1^H and ^15^N spectra were observed for a number of the assigned residues (see Supplementary Fig. S8 for examples of specific residue chemical shift perturbations during the titration). Since shifts can be influenced by through-bond, through-space and allosteric interactions, it is difficult to determine whether the CSPs in IdmH are a result of direct binding or conformational change. However, to localize the protein–ligand interactions, the geometrical distance moved by each peak was calculated for all assigned residues [Fig. 6 ▸(*a*)] and chemical shifts larger than the standard deviation of all shifts were mapped onto the IdmH diagram using yellow, orange and red colours [Fig. 6 ▸(*b*)].

In the presence of indanomycin (the bound spectrum), some resonances moved a substantial distance from their resonance counterparts in the native protein (the free spectrum) making it impossible to match the two accurately. For this reason, a ‘minimum chemical shift procedure’ was employed (Farmer *et al.*, 1996 ▸; Lüttgen *et al.*, 2002 ▸; Williamson, 2013 ▸). This method links each assigned free resonance to the signal in the bound spectrum that has moved the least from the position in the free spectrum. Therefore, each resonance is assigned a CSP, and while the true change might be larger than that assigned, it will never be smaller.

The results of chemical shift mapping suggest that the most significant CSPs are localized at the top of the hydrophobic cavity of IdmH (α-helices 3–4) and on the β-sheet inside the pocket (β-strands 2–4) [Figs. 2 ▸ and 6 ▸(*c*)], consistent with the predicted location of the IdmH active site. A closer look at the active-site residues [Fig. 6 ▸(*d*)] revealed that the majority of the resonances exhibited a moderate (Val62 and Met115) to large (Trp59, Trp63 and Tyr16) shift. Two resonances, Ser133 and Ile37, exhibited a shift below the level of significance (<0.72 p.p.m.) and three remaining resonances, Thr131, Phe19 and His129, do not have their backbone resonances assigned. These results thus suggest that indanomycin can bind to IdmH in the proposed active site, and that binding, and potentially catalysis, could induce conformational changes which spread to other areas of the enzyme, as highlighted by the more distal CSPs.

In a bid to obtain further details of indanomycin binding to IdmH, we performed both co-crystallization and crystal-soaking experiments to try and determine a structure of the product complex. Co-crystallization with the ligand appeared to inhibit crystal formation, likely owing to the high concentration of DMSO required to keep the ligand in solution, whilst crystal soaking did not result in significant new electron density that could be confidently attributed to the ligand. In the light of these results, we therefore elected to explore computational methods as a means of further analyzing the biochemical function of IdmH.

### 3.4. Docking the product and simulating the protein–product complex   

**Figure 7:**
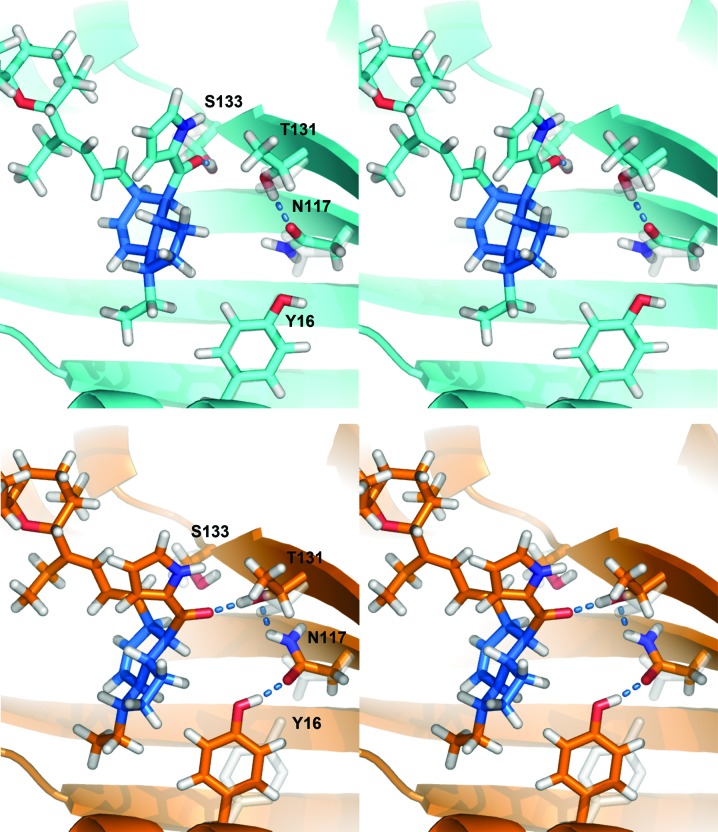
Stereoview of the position and interactions of indanomycin docked in the wild-type IdmH chain *B* active site after brief structural optimization. Top: pose C, indanomycin C21=O hydrogen-bonds to Ser133. Bottom: pose D, indanomycin C21=O hydrogen-bonds to Thr131. The Tyr16, Asn117 and Thr131 side-chain positions from the original crystal structure are shown transparently in light grey.

**Figure 8:**
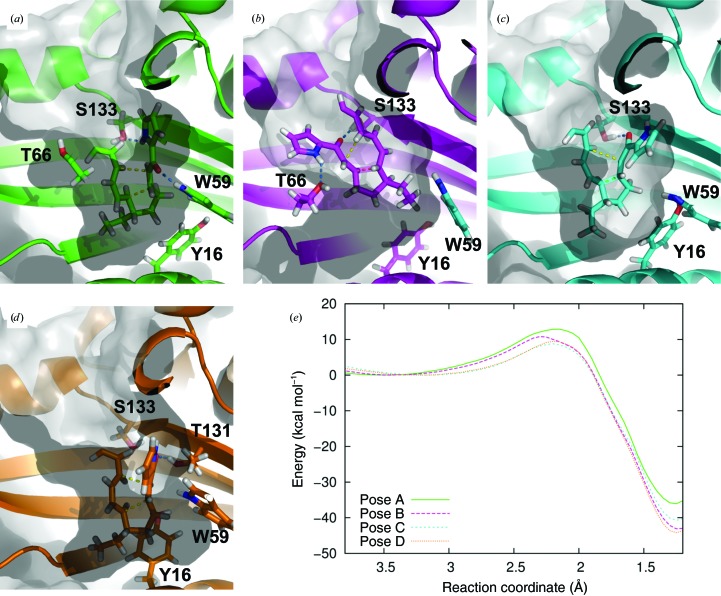
The results of approximate QM/MM reaction simulations (DFTB2/ff14SB). (*a*)–(*d*) show representative (approximate) transition-state structures of the four possible enzyme–product complexes (obtained by clustering) together with their energy profiles in (*e*). Only part of the indanomycin structure is shown in these panels for clarity, but the remainder of the indanomycin molecule (including the tetrahydropyran ring, which may or may not be cyclized before IdmH-catalysed formation of the indane ring), was present in all simulations.

Having determined that indanomycin binds in the active site of IdmH, we wanted to further explore the likely chemical reaction catalysed by IdmH. Indanomycin was placed in the wild-type IdmH active sites (chain *A* and *B*) using *in silico* molecular docking. Three significantly different binding poses were found (two in chain *A* and one in chain *B*). For the binding pose in chain *B*, the conformer for Asn117 was adjusted to provide a slight difference in the hydrogen-bonding environment. This led to the following four options: pose A, the indane ring bound in the chain *A* pocket but without any specific hydrogen bonds being made; pose B, the indane ring in the chain *A* pocket with a hydrogen bond formed between Ser133 and the substrate carbonyl group (C21=O); pose C, the indane ring in the chain *B* pocket with a hydrogen bond formed between Ser133 and the substrate carbonyl group (C21=O) but in a different orientation to pose B (Fig. 8); pose D, the indane ring in the chain *B* pocket with a hydrogen bond formed between Thr131 and the substrate carbonyl group in which a new hydrogen-bond network is formed in the protein between Asn117 and Tyr16. Poses C and D are shown in Fig. 7 ▸ (after brief structural optimization), with the main difference in binding pose arising from the side-chain orientation of Asn117. Subsequently, brief structural optimization and 100–300 ps molecular-dynamics simulation was performed for each of the four poses.

### 3.5. QM/MM simulations of the reverse reaction   

11 QM/MM reaction simulations (starting from independent snapshots of the molecular-dynamics simulations) were run for all four possible enzyme–substrate complexes using the same approximate protocol as described in Byrne *et al.* (2016 ▸). Representative (approximate) transition-state structures of these four complexes are shown in Fig. 8 ▸, together with the free-energy profile obtained from the 11 simulations together. Based on the energy barriers, reactions proceeding from starting poses C and D are the most likely (Fig. 7 ▸). The low barriers compared with poses A and B can be rationalized based on the electron-withdrawing nature of the hydrogen bond formed between Ser133 or Thr131 and the substrate carbonyl at C21 (depending on the pose). Between the two, pose D seems to be the most likely, based on a good fit with the cavity and some possible catalytic stabilization by the positioning of Trp59. The reaction barrier for option A is highest, which could be expected owing to the lack of a (good) hydrogen-bonding interaction in this pose; during the QM/MM simulations a hydrogen bond to Trp59 can be formed (see Fig. 8 ▸), but this interaction is more transient and thus does not offer the same benefit as the hydrogen bonds formed in poses C and D. MD and QM/MM simulation of option B lead to a poorer fit with the active-site cavity, suggesting that this is an unlikely starting point for catalysis by IdmH (Fig. 8 ▸).

The chemical shift mapping results (Fig. 6 ▸) further support the observations from the approximate QM/MM simulations. Pose D involves a hydrogen-bonding network between Thr131, Asn117 and Tyr16 as well as catalytic stabilization from Trp59. According to the NMR titration experiments, all of these residues (except for Thr131, which could not be assigned) are involved in protein–ligand interactions as measured by significant CSPs (0.22 p.p.m. for Tyr16, 0.12 p.p.m. for Asn117 and 0.20 p.p.m. for Trp59). There are also van der Waals interactions between the substrate and the other residues with significant CSPs. It is further worth noting that Ser133, which is involved in a hydrogen bond to the substrate in pose C, displayed a CSP (0.012 p.p.m.) below the level of significance (0.07 p.p.m.), making pose C less likely than pose D. The reaction simulations indicate that an asynchronous concerted mechanism is followed, with the formation of the C15–C19 bond occurring first (and representing the reaction barrier; see Fig. 8 ▸); this is similar to the situation in the intramolecular Diels–Alder reaction in AbyU and indeed the uncatalysed reaction (Byrne *et al.*, 2016 ▸).

In summary, chemical shift perturbation mapping, *in silico* docking and approximate QM/MM simulations reveal the structure of the likely reactive IdmH–substrate complex and show that the active-site shape, along with a critical hydrogen bond, is likely to promote the correct conformation of the substrate **4** (bringing together the diene and dienophile) and provides an appropriate environment for the indane-ring cyclization reaction to occur.

## 4. Discussion   

The Diels–Alder reaction is of major synthetic value in organic chemistry for the preparation of substituted six-membered rings with the creation of up to four new stereocenters (Stocking & Williams, 2003 ▸). The potential use of proteins to catalyse Diels–Alder reactions stereoselectively under mild conditions therefore remains of high interest. Until recently, no natural enzymes that specifically catalyse Diels–Alder reactions (*i.e.* Diels–Alderases) were known (Jamieson *et al.*, 2019 ▸), and some progress was made with obtaining catalytic antibodies and *de novo* designed enzymes that catalyse a bimolecular Diels–Alder reaction (Gouverneur *et al.*, 1993 ▸; Siegel *et al.*, 2010 ▸). During the late 1990s and early 2000s, three putative Diels–Alderases involved in natural product synthesis were reported: macrophomate synthase (Watanabe *et al.*, 2000 ▸), lovastatin nonaketide synthase (LovB; Auclair *et al.*, 2000 ▸) and solanapyrone synthase (Sol5; Katayama *et al.*, 1998 ▸; Kasahara *et al.*, 2010 ▸). LovB and Sol5 have activities other than catalysing a Diels–Alder reaction, which makes proving any catalysis of the cyclo­addition reaction difficult. The bimolecular reaction catalysed by macrophomate synthase is now thought to follow a stepwise mechanism: Michael addition followed by an aldol reaction (Guimarães *et al.*, 2005 ▸). In the 2010s, the first enzyme specifically catalysing only a Diels–Alder reaction was reported: SpnF, which is involved in the biosynthesis of spinosyn A (Kim *et al.*, 2011 ▸). Subsequently, numerous standalone Diels–Alderases have been identified, especially from bacterial biosynthesis pathways, including the cofactor-free PyrI4 (Zheng *et al.*, 2016 ▸) from the pyrroindomycin biosynthetic pathway and its homologues AbyU (Byrne *et al.*, 2016 ▸) and VstJ (Hashimoto *et al.*, 2015 ▸) from other spiro­tetronate-containing natural products.

Here, we propose that IdmH from the biosynthesis of indanomycin is another natural standalone Diels–Alderase. Similar to the structurally characterized natural Diels–Alderases AbyU (Byrne *et al.*, 2016 ▸) and PyrI4 (Zheng *et al.*, 2016 ▸), the enzyme acts in the (final) tailoring steps of natural product biosynthesis, does not require a cofactor and the reaction catalysed is intermolecular. The IdmH structure is a homodimeric ‘barrel’ that contains a ‘capping loop’. With the capping loop closed, a cavity is formed that fits the substrate in a conformation in which the diene and dienophile moieties are positioned in line with a Diels–Alder reaction. Interestingly, the structure and sequence of IdmH are substantially different from those of AbyU and PyrI4 (as well as their homologues from other spirotetronate and spirotetramate biosynthesis pathways; Supplementary Fig. S9). Instead of a β-barrel, IdmH is an α+β barrel. There is no significant sequence similarity between IdmH on the one hand and AbyU and PyrI4 on the other, nor is there an obvious mapping of the four antiparallel β-strands to the four antiparallel β-strands of the AbyU and PyrI4 β-barrels. Despite the absence of a clear evolutionary relationship between the IdmH α+β barrel and the β-barrels of AbyU and PyrI4, the overall shape is similar, with the substrate cavity near the capping loop in each case (Supplementary Fig. S9; Hashimoto & Kuzuyama, 2016 ▸). This raises the question of whether the emergence of these intramolecular Diels–Alderases with similar structures provide an example of convergent evolution. Further, it indicates that not only a β-barrel scaffold, but also a α+β barrel scaffold, could be used for the design of engineered or *de novo* designed Diels–Alderases, thereby potentially allowing a broader range of substrates.

## 5. NMR data   

The NMR assignment data for IdmH have been deposited in the Biological Magnetic Resonance Data Bank (BioMagResBank) with accession number 27838.

## Supplementary Material

PDB reference: IdmH, truncated (Δ99–107), selenomethionine-containing, 6hnl

PDB reference: truncated (Δ99–107), wild type, 6hnm

PDB reference: full length, wild type, 6hnn

Supplementary Figures and Tables. DOI: 10.1107/S2052252519012399/tj5026sup1.pdf

## Figures and Tables

**Table 1:** Data-collection and refinement statistics Values in parentheses are for the highest resolution shell.

	Selenomethionine-labelled IdmH-Δ99–107	Native IdmH-Δ99–107	Wild-type IdmH
Data collection			
Wavelength (Å)	0.966	0.976	0.9795
Space group	*F*23	*F*23	*P*12_1_1
*a*, *b*, *c* (Å)	152.6, 152.6, 152.6	152.7, 152.7, 152.7	66.7, 103.5, 99.6
α, β, γ (°)	90.0, 90.0, 90.0	90.0, 90.0, 90.0	90.0, 91.6, 90.0
Resolution (Å)	76.3–2.2 (2.27–2.20)	76.3–2.0 (2.05–2.00)	51.8–2.7 (2.82–2.70)
*R*_merge_	0.118 (4.620)	0.046 (0.669)	0.086 (0.961)
*R*_meas_	0.120 (4.697)	0.053 (0.769)	0.097 (1.098)
*R*_p.i.m_	0.022 (0.846)	0.026 (0.373)	0.046 (0.522)
〈*I*/σ(*I*)〉	20.8 (1.4)	12.7 (1.4)	11.4 (1.4)
CC_1/2_	0.999 (0.812)	0.999 (0.516)	0.998 (0.656)
Completeness (%)	100 (100)	99.9 (99.9)	99.6 (99.5)
Multiplicity	30.6 (30.6)	4.1 (4.1)	4.5 (4.3)
Refinement			
Resolution (Å)	88.1–2.2	88.2–2.0	103.5–2.7
No. of reflections (total/free)	14989/726	19980/974	37126/1832
*R*_work_/*R*_free_ †	0.212/0.276	0.230/0.271	0.211/0.245
Total No. of atoms	3707	3704	19690
No. of ligands	0	0	0
No. of water molecules	13	27	9
Wilson *B* factor (Å^2^)	48.3	48.4	60.7
R.m.s. deviations			
Bond lengths (Å)	0.016	0.007	0.012
Bond angles (°)	1.81	1.15	1.47
Ramachandran plot			
Favoured (%)	98.8	98.8	98.46
Allowed (%)	1.2	1.2	1.47
Outliers (%)	0	0	0.07
Molecules in asymmetric unit	2	2	10
PDB code	6hnl	6hnm	6hnn

† 5% of the data was set aside for *R*_free_ calculations.
