# Supplementary material for: Crystal structure of the putative cyclase IdmH from the indanomycin nonribosomal peptide synthase/polyketide synthase
Source: IUCrJ. 2019 Oct 24;6(Pt 6):1120–33. doi: 10.1107/S2052252519012399 (PMC6830212; doi:10.1107/S2052252519012399)
Supplement: Supplementary file 1 [file m-06-01120-sup1.pdf]

IUCrJ

Volume 6 (2019)

Supporting information for article:

Crystal structure of the putative cyclase IdmH from the indanomycin NRPS/PKS

Ieva Drulyte, Jana Obajdin, Chi H. Trinh, Arnout P. Kalverda, Marc van der Kamp, Glyn R. Hemsworth and Alan Berry

```
                               10         20         30         40         50
This paper (WT)        GSHMAHQPSD TIAGLYEAFN SGDLETLREL IAPDAVIHLP GTAGDAEHPP
This paper (Δ99-107)   GSHMAHQPSD TIAGLYEAFN SGDLETLREL IAPDAVIHLP GTAGDAEHPP
ACN69984.1                MAHQPSD TIAGLYEAFN SGDLETLREL IAPDAVIHLP GTAGDAEHPP

                               60         70         80         90        100
This paper (WT)        GTPRDREGWL GVWQFTQAFF PDMTATVQDI VQTGDLVATR CVARGTHSIE
This paper (Δ99-107)   GTPRDREGWL GVWQFTQAFF PDMTATVQDI VQTGDLVATR CVARGTHS--
ACN69984.1             GTPRDREGWL GVWQFTQAFF PDMTATVQDI VQTGDLVATR CVARGTHSIE

                              110        120        130        140
This paper (WT)        FMGVPPTGRP FEMTMLNMSR VRDGRIVEHW TISDNVTMLA QLGVKASL
This paper (Δ99-107)   -------GRP FEMTMLNMSR VRDGRIVEHW TISDNVTMLA QLGVKASL
ACN69984.1             FMGVPPTGRP FEMTMLNMSR VRDGRIVEHW TISDNVTMLA QLGVKASL
```

Figure S1 Protein sequences and numbering of IdmH. IdmH is predicted to be a 145-residue protein from the Uniprot database (ACN69984.1). During the cloning and expression of IdmH reported in this paper, a hexa-histidine tag for purification followed by a thrombin cleavage site was added. Following purification on a 5 ml HisTrap HP column (GE Healthcare, USA), thrombin cleavage produced a ‘wild-type’ IdmH protein of 148 residues (listed above). Throughout this paper numbering of residues relates to this thrombin-cleaved wild-type sequence. The Δ99-107 variant was also constructed. NMR data were collected for IdmH that had not undergone thrombin cleavage and hence that protein contains an additional N-terminal sequence of GSSHHHHHHGLVPR.

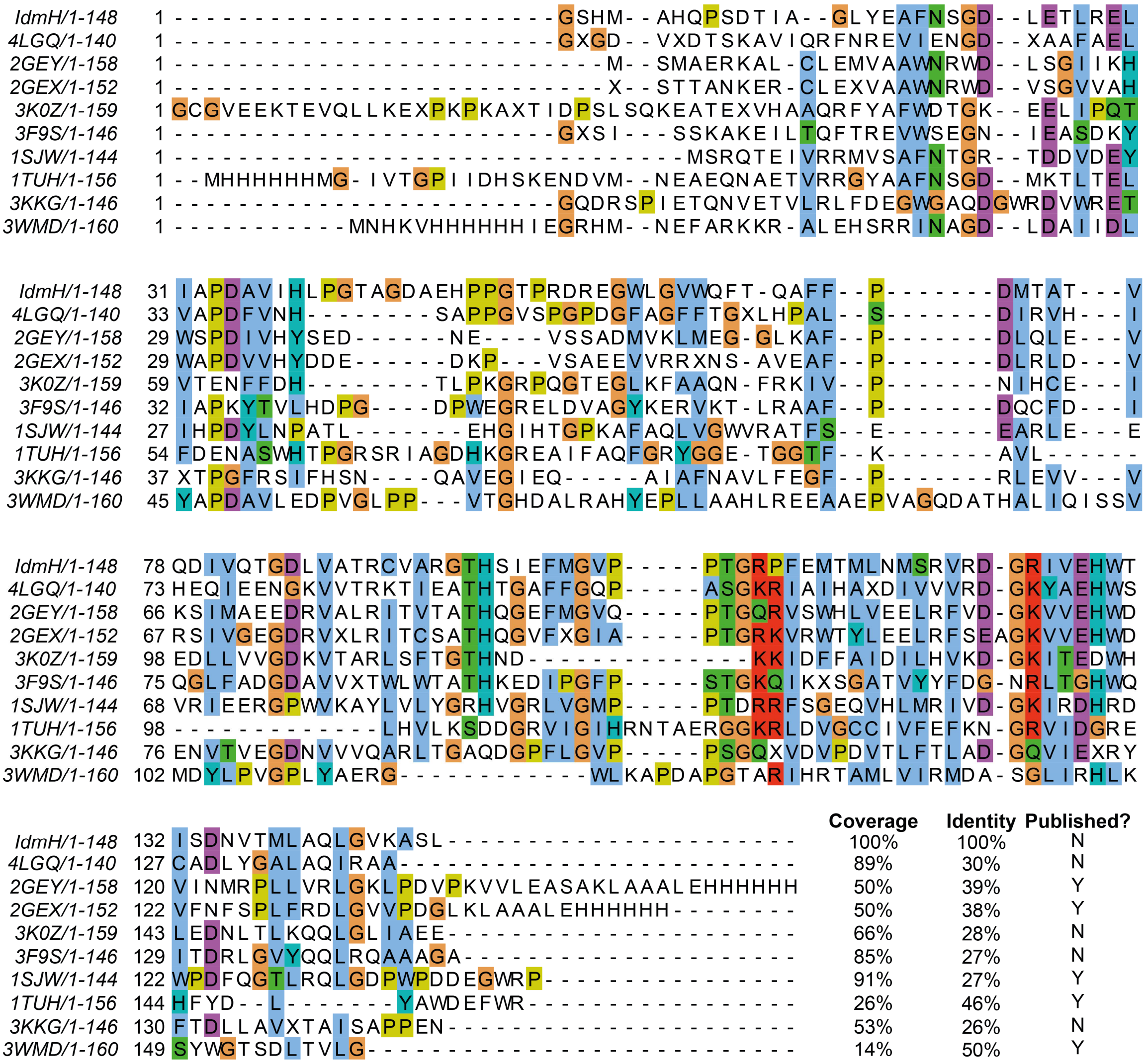

Figure S2 Sequence alignment of IdmH to its homologs. The sequence of IdmH was searched against the PDB database. Conserved residues are highlighted in colour using Jalview software and Clustal scheme. The associated query coverage and sequence identities with IdmH are also included.

4LGQ: putative polyketide cyclase from *Chromobacterium violaceum*; 2GEY: AclR putative hydroxylase from *Streptomyces galilaeus* [Beinker et al 2006 JMB 359, 728-40]; 2GEX: SnoaL2 putative hydroxylase from *Streptomyces nogalater* [Beinker et al 2006 JMB 359, 728-40]; 3K0Z: putative polyketide cyclase from *Bacillus cereus* ATCC10987; 3F9S: double mutant of N-lobe human transferrin [Mason et al 2009 Biochem 48, 1945-53]; 1SJW: SnoaL polyketide cyclase [Sultana et al 2004 EMBO J 23, 1911-21]; 1TUH: Bal32a member of the adaptable a + b barrel family [Robinson et al JMB 2005 346, 1229-41]; 3KKG: putative SnoaL-like polyketide cyclase from *Jannaschia* sp CCS1; 3WMD: epoxide hydrolase MonB1 [Minami et al ACS Chem Biol 2014, 9, 562-9].

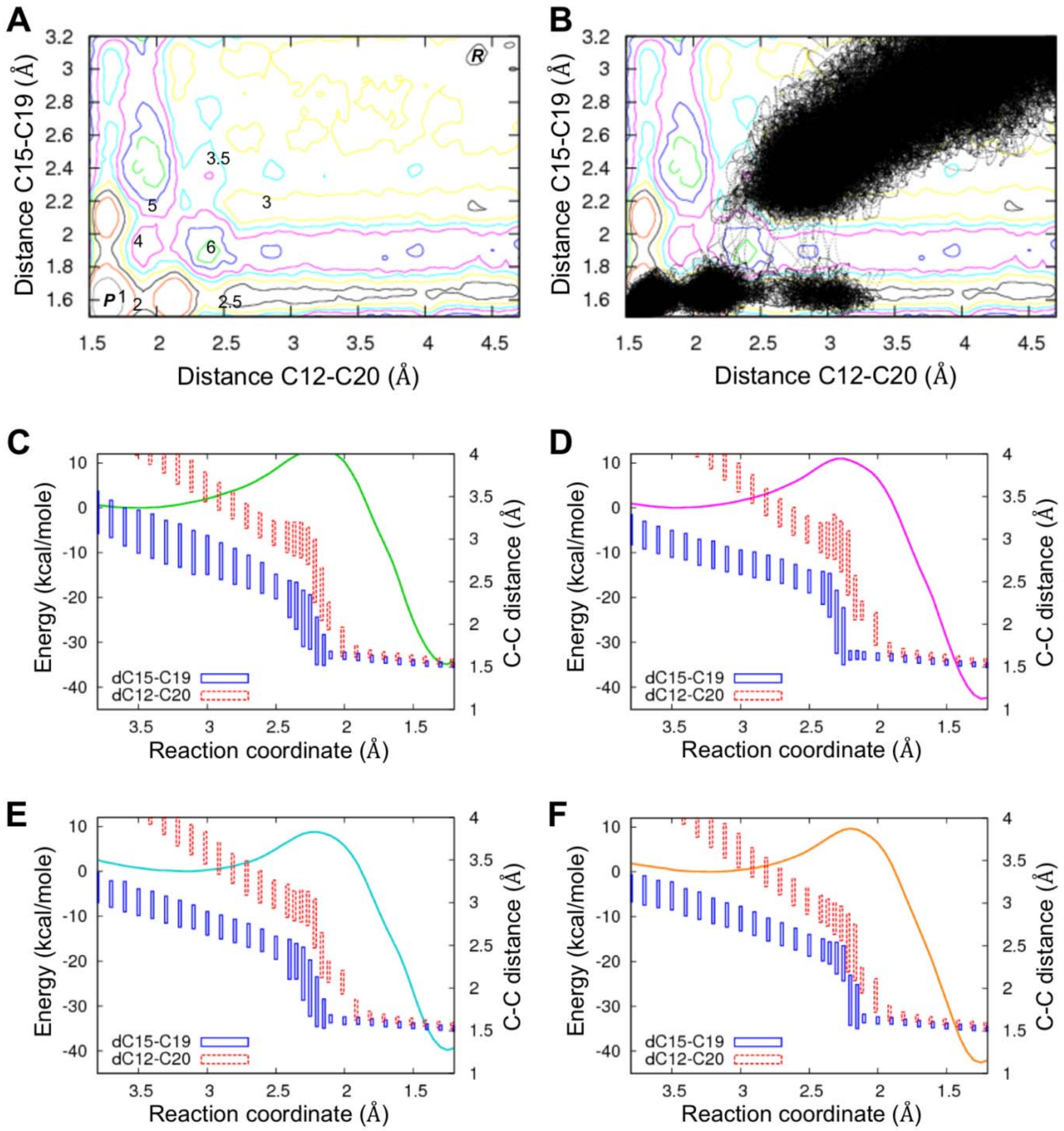

Figure S3 Analysis of reaction simulations using the approximate 1D reaction coordinate. A) Approximate 2D PMF for pose D, calculated using the carbon-carbon distances as the reaction coordinates for umbrella sampling, with the same simulation details as the 1D umbrella sampling. Contour lines and labels indicate energies in kcal/mole. Reactant (R) and Product (P) minima are indicated. B) Progression of the carbon-carbon distances in the 11 1D-PMF runs for pose D projected on the 2D PMF from panel A. C-F) 1D PMF for poses A, B, C & D (green, magenta, cyan and orange, respectively, to match with Figure 8), with the range of carbon-carbon distances sampled in each window indicated by the average +/- standard deviation.

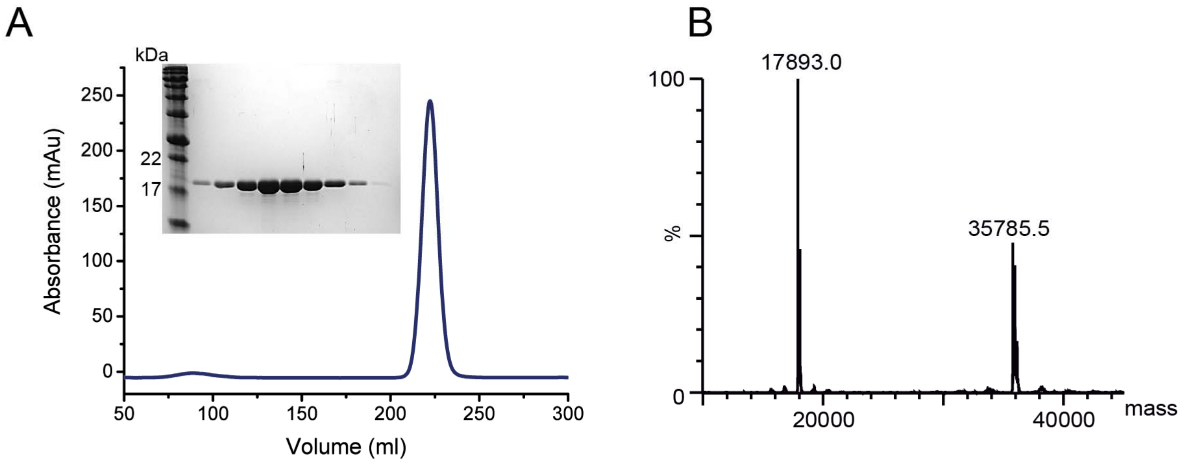

Figure S4 Purification and characterisation of wild-type IdmH. *(a)* Purification of IdmH by size-exclusion chromatography. Peak fractions were analysed by SDS-PAGE which shows a single monomeric band for denatured IdmH. *(b)* Analysis of IdmH oligomerisation state by native liquid chromatography / mass spectrometry. A clear peak for dimeric IdmH indicates the association on IdmH into homo-dimers.

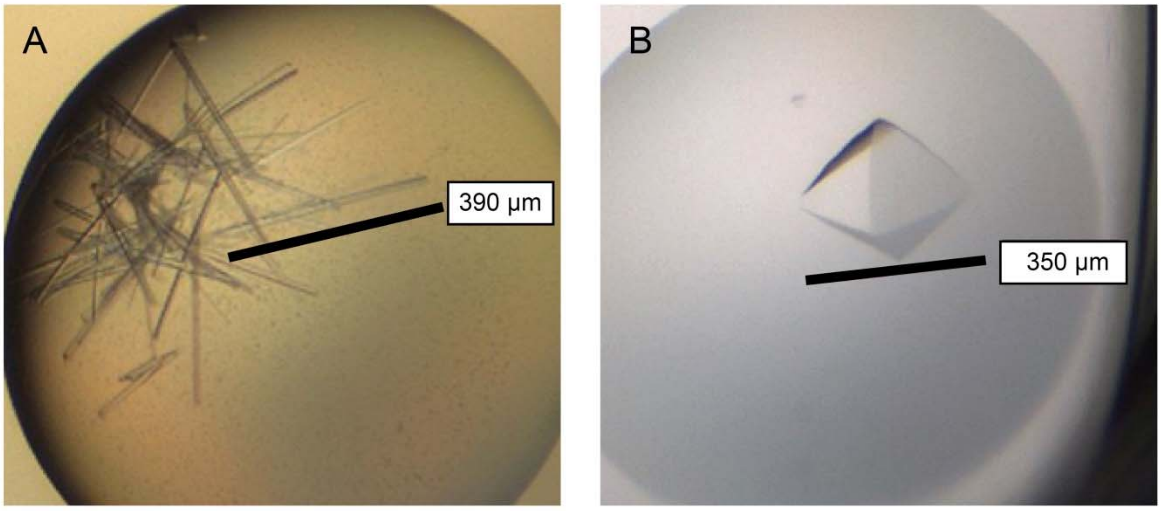

Figure S5 Crystal morphology of IdmH and IdmH-Δ99-107. *(a)* Wild-type IdmH crystallised as long rods in the space group $P12_12$. *(b)* IdmH-Δ99-107 resulted in large tetrahedral crystals with a space group *F*23.

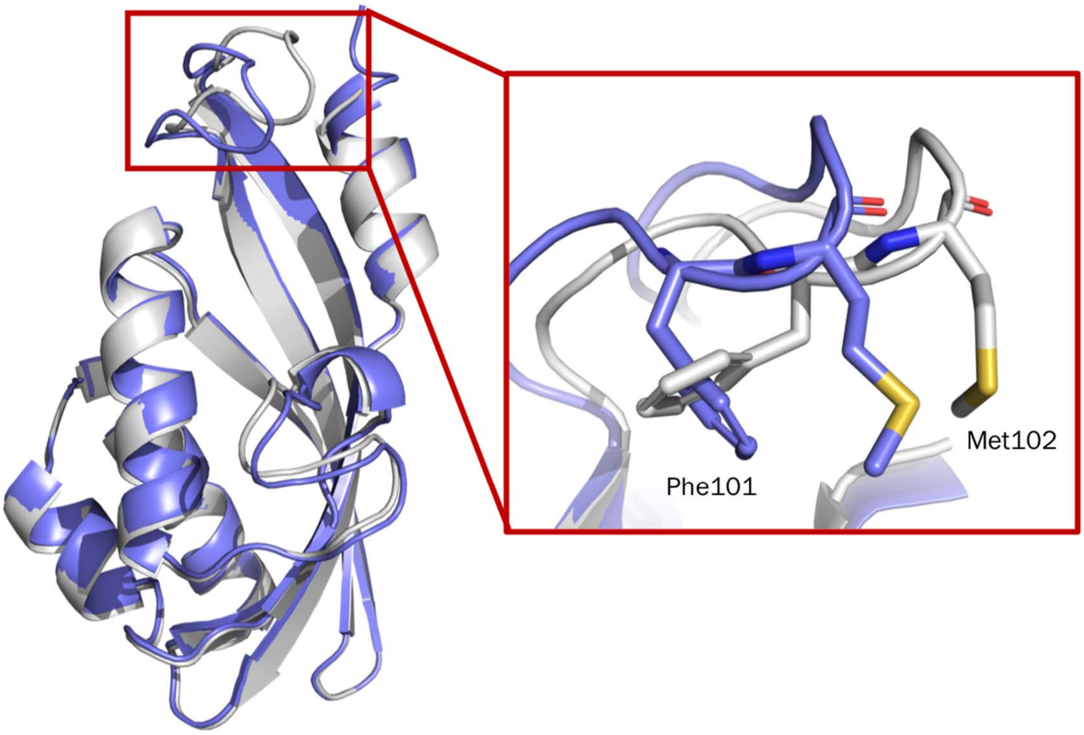

Figure S6 Conformational differences in the loop comprising residues 99-108. This loop was truncated in IdmH-Δ99-107 to aid crystallisation while in the wild-type IdmH structure it has been observed in two distinct conformations between chains A (blue) and B (grey). The inset shows a magnified view of the loop and two amino acids Phe101 and Met102 are shown as sticks.

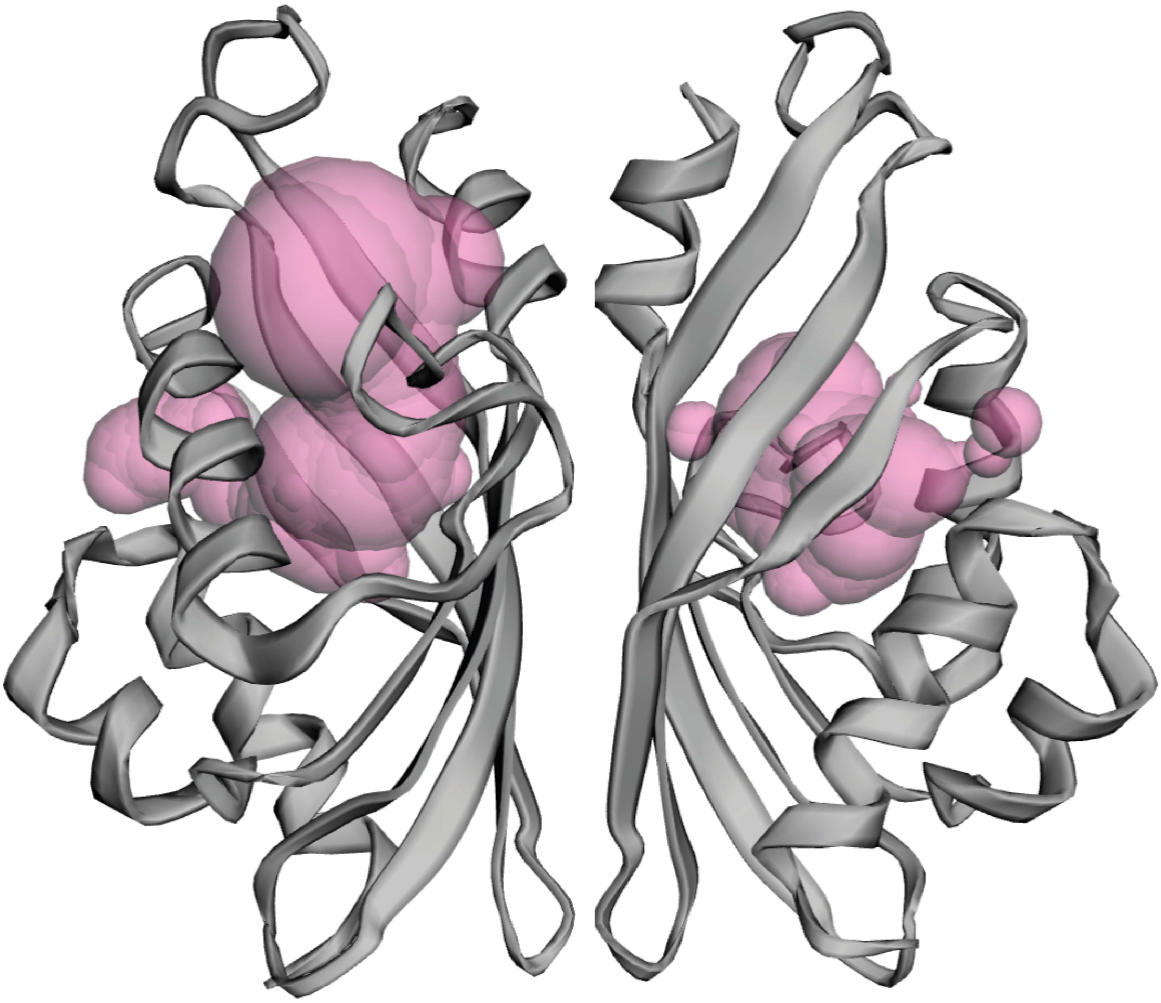

Wild-type IdmH Chain A
Solvent-accessible surface area: 464 Å^2
Solvent-accessible volume: 410 Å^3

Wild-type IdmH Chain B
Solvent-accessible surface area: 301 Å^2
Solvent-accessible volume: 234 Å^3

Figure S7 The solvent-accessible volume (pink) of hydrophobic pockets from IdmH chains A and B, as computed by CASTp 3.0 (Tian *et al* (2018)). The CASTp server describes a pocket as an empty concavity on the protein surface into which solvent with a probe radius of 1.4 Å can gain access. In such pockets the mouth opening of the pocket needs to be smaller than the cross-section of the pocket itself. If the mouth is wider than the pocket cross-section, the cavity is labelled as a shallow depression and is excluded from the solvent-accessible surface area and volume calculations. In chain B of IdmH, the loop capping the pocket is in a more open conformation than in chain A, resulting in part of the chain B pocket becoming labelled as a shallow depression. The volume is therefore excluded from the calculation and is excluded from the area and volume calculations. The pocket in chain B therefore appears smaller on the figure above. This figure however highlights the location of the active site pocket.

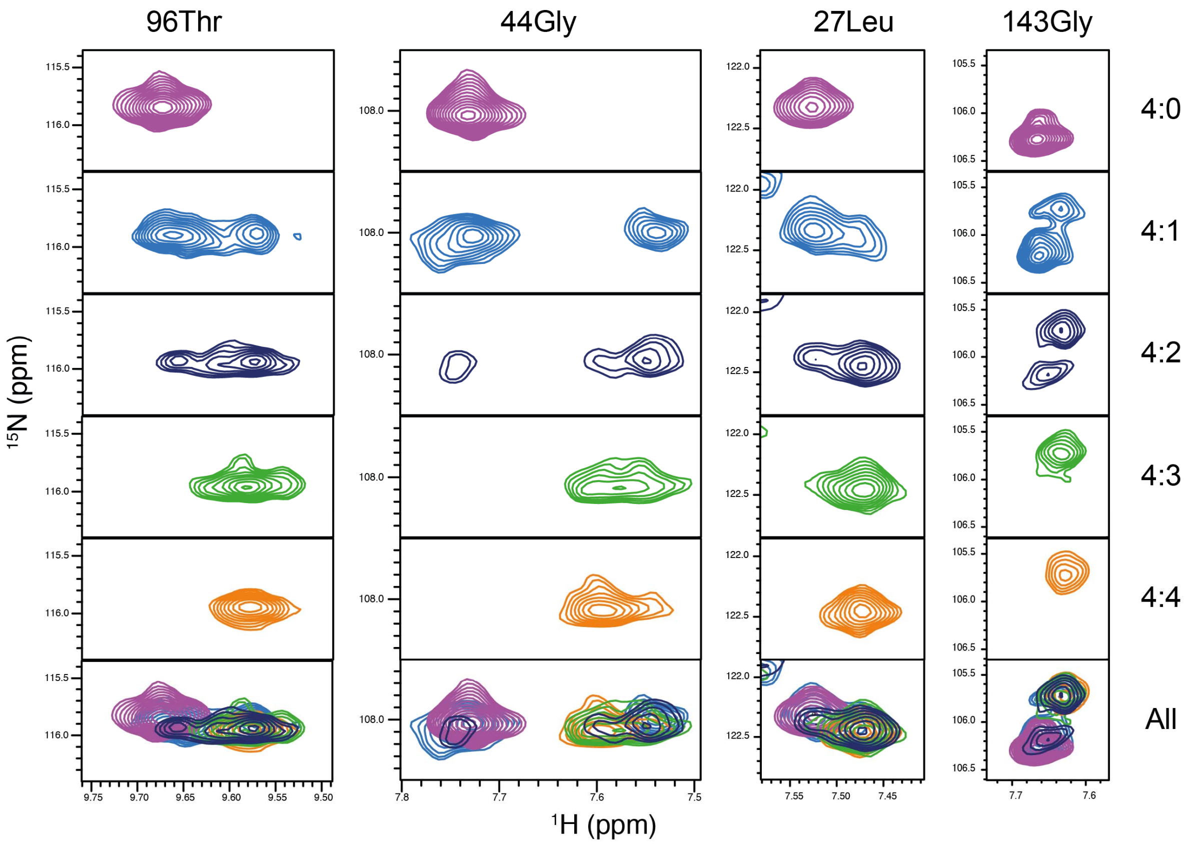

Figure S8 Chemical shift perturbations for individual residues going from free (purple) to bound (orange) state as recorded in ^{1}H–^{15}N HSQC-TROSY spectra. Peak broadening is observed when in equilibrium between free and bound (4:2 and 4:3 ratios of protein: ligand, dark blue and green, respectively) states. Peaks sharpen up again close to saturation (orange). In all cases, the free peaks decrease in intensity as the bound peaks increase, suggesting a slow exchange.

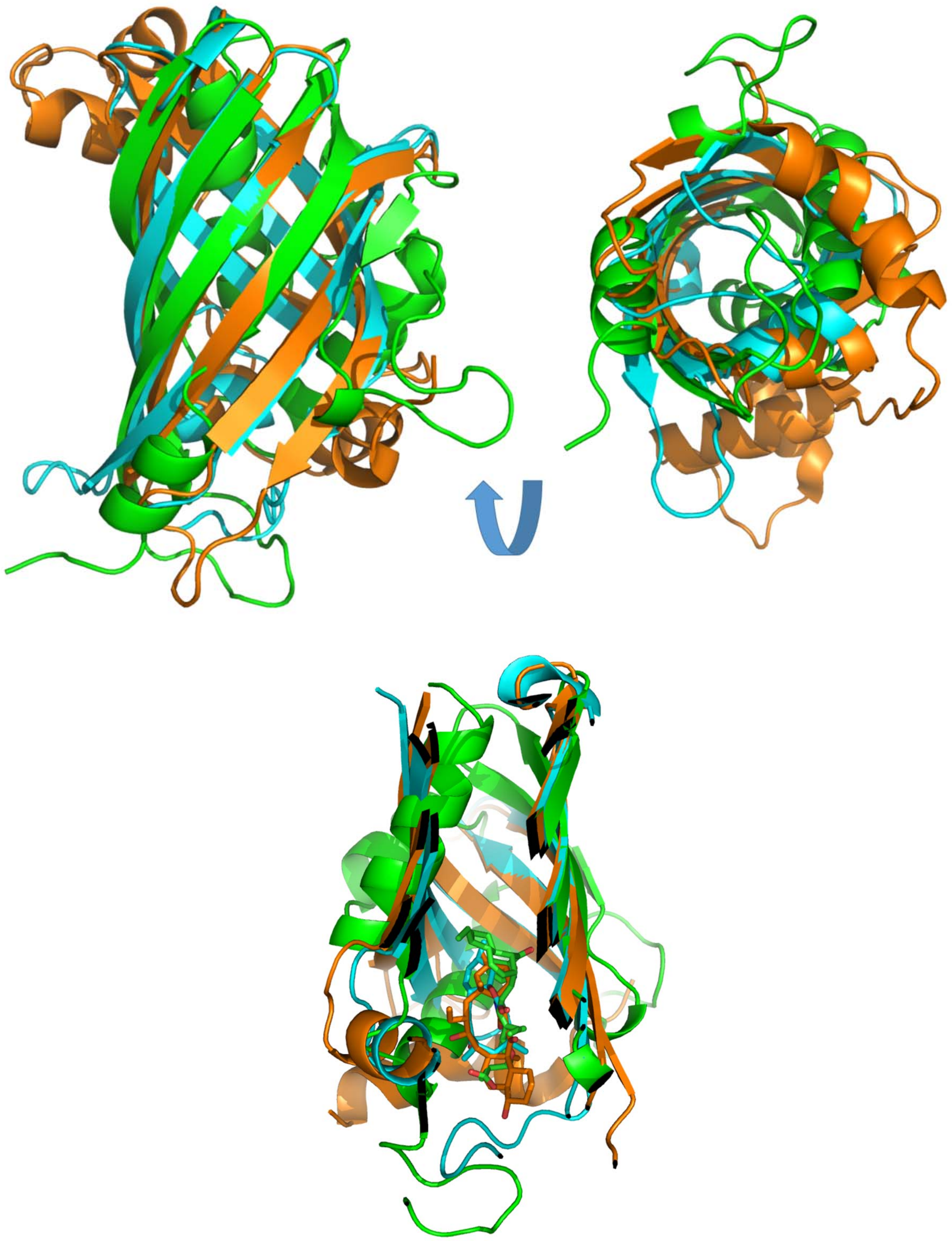

Figure S9 Comparison of monomers of IdmH (chain A; green), AbyU (PDB 5DYV, chain A; cyan) and PyrI4 (PDB 5BU3, chain A; orange). AbyU and PyrI4 were aligned based on Cα RMSD; IdmH was manually positioned to indicate similarity in structure. The bottom panel shows the location of the products, either co-crystallised (Pyrl4) or docked (AbyU from (Byrne *et al* (2016)) and IdmH (this work; pose D)).

Table S1 IdmH variants produced and the outcome.

**Variant**	**Justification**	**Outcome**
E29A	Reducing surface entropy	No effect
E47A	Reducing surface entropy	No effect
E57A	Reducing surface entropy	No effect
E100A	Reducing surface entropy	No effect
Residue 49-53 truncation	Decreasing flexibility	Insoluble
Residue 99-107 truncation	Decreasing flexibility	New crystal form
Residue 142-147 truncation	Decreasing flexibility	Insoluble

Table S2 NMR peak assignment table.

NMR peaks were assigned by labelling IdmH with ^{13}C, ^{15}N and ^{2}H, and the subsequent collection and processing of a number of triple resonance spectra (Yamazaki *et al* (1994)). Here, for each backbone nucleus we report residue number, amino acid type and chemical shift values for H, N, C, CA and CB chemical groups. This data is also deposited in the Biological Magnetic Resonance Data Bank (BioMagResBank) with accession number 27838.

Residue No.	Amino acid	H (ppm)	N (ppm)	C (ppm)	CA (ppm)	CB (ppm)
-4	Gly	-	-	174.04	45.33	-
-3	Leu	7.90	121.86	177.29	55.20	41.84
-2	Val	7.92	122.62	174.59	59.80	32.31
-1	Pro	-	-	177.09	63.14	31.70
0	Arg	8.32	122.19	-	56.42	30.39
1	Gly	8.34	110.56	-	45.38	-
2	Ser	-	-	-	-	-
3	His	-	-	175.48	56.69	30.37
4	Met	7.93	121.51	175.65	55.40	32.65
5	Ala	8.06	124.98	177.43	52.32	18.92
6	His	8.13	119.59	175.81	56.37	30.84
7	Gln	8.72	122.81	-	54.23	28.64
8	Pro	-	-	178.02	66.57	31.66
9	Ser	8.23	110.83	176.28	62.13	62.34
10	Asp	7.60	123.05	179.66	57.50	40.58
11	Thr	7.66	119.96	175.24	67.06	68.63
12	Ile	7.37	120.06	177.29	61.51	35.64
13	Ala	7.98	123.20	181.59	55.66	17.61
14	Gly	7.87	105.10	175.79	46.51	-
15	Leu	7.82	125.58	177.58	58.15	41.43
16	Tyr	7.16	114.80	178.17	64.72	36.93
17	Glu	7.56	119.58	179.11	59.68	29.09
18	Ala	7.86	123.09	180.63	54.99	16.24
19	Phe	-	-	179.73	61.70	38.04
20	Asn	8.16	118.23	177.36	55.81	37.37
21	Ser	7.89	113.21	175.29	58.51	64.67
22	Gly	7.97	113.72	174.57	46.96	-

23	Asp	8.07	119.52	175.24	52.31	39.22
24	Leu	7.77	125.86	178.52	57.68	41.40
25	Glu	7.99	117.65	180.08	59.66	28.57
26	Thr	7.33	118.49	176.72	66.44	66.94
27	Leu	7.52	121.95	178.09	58.79	40.57
28	Arg	7.46	113.79	176.75	59.78	29.00
29	Glu	7.14	117.51	179.07	58.20	29.75
30	Leu	7.57	117.25	175.74	56.10	43.06
31	Ile	7.25	118.06	175.23	60.18	39.99
32	Ala	8.37	130.10	177.05	51.66	17.26
33	Pro	-	-	177.79	65.26	30.70
34	Asp	8.36	115.01	175.81	51.93	38.52
35	Ala	7.28	122.38	176.03	52.73	20.86
36	Val	7.20	120.34	173.99	62.23	32.01
37	Ile	8.52	126.60	174.87	57.93	35.08
38	His	9.03	128.63	174.65	53.67	26.98
39	Leu	7.73	125.01	174.92	52.28	40.30
40	Pro					
41	Gly	-	-	175.86	48.02	-
42	Thr	7.40	111.56	174.97	63.30	68.94
43	Ala	7.99	123.86	178.37	51.88	19.58
44	Gly	7.75	107.81	172.24	45.30	-
45	Asp	8.03	121.11	176.72	53.33	41.48
46	Ala	8.19	120.72	179.41	55.27	18.20
47	Glu	7.72	114.63	175.88	57.15	29.95
48	His	7.81	117.86	171.78	53.23	28.51
49	Pro					
50	Pro	-	-	176.73	64.33	32.05
51	Gly	9.15	114.26	175.36	45.24	-
52	Thr	8.05	117.48	173.91	60.49	69.73
53	Pro	-	-	175.55	63.02	32.60
54	Arg	8.66	117.47	175.00	51.82	32.01
55	Asp	7.63	119.65	176.88	51.43	41.42
56	Arg	-	-	177.99	61.10	30.60
57	Glu	8.39	116.31	180.62	58.95	28.00

58	Gly	8.61	111.15	175.95	47.11	-
59	Trp	8.38	124.21	177.96	58.30	29.66
60	Leu	8.73	117.42	180.22	58.62	39.48
61	Gly	8.21	107.43	176.98	47.38	-
62	Val	7.94	125.70	179.61	66.65	31.66
63	Trp	8.94	124.39	178.19	61.84	27.65
64	Gln	8.47	118.74	178.85	59.40	29.01
65	Phe	8.00	121.18	177.38	60.90	38.87
66	Thr	7.94	115.12	176.78	66.75	69.10
67	Gln	8.13	122.32	177.19	57.42	27.47
68	Ala	6.92	117.93	179.54	53.91	17.46
69	Phe	6.89	114.37	174.21	59.41	40.02
70	Phe	7.11	113.89	173.76	54.48	40.53
71	Pro	-	-	178.28	65.19	31.42
72	Asp	7.78	115.77	176.56	52.32	40.14
73	Met	8.02	122.13	177.47	56.95	33.56
74	Thr	8.81	118.59	173.54	60.01	73.56
75	Ala	9.47	124.58	176.80	51.05	20.65
76	Thr	8.27	117.87	175.11	61.09	70.65
77	Val	9.02	129.47	175.80	64.05	31.61
78	Gln	9.01	126.59	174.91	55.92	30.03
79	Asp	7.05	115.50	174.43	54.20	46.86
80	Ile	8.41	120.33	173.61	60.33	41.03
81	Val					
82	Gln	-	-	176.30	54.45	32.36
83	Thr	8.64	122.20	174.24	63.99	73.92
84	Gly	-	-	174.23	47.69	-
85	Asp	8.48	126.47	174.29	53.77	40.35
86	Leu	7.81	119.74	176.27	45.95	-
87	Val	-	-	175.57	59.67	35.40
88	Ala	9.78	133.37	176.11	50.19	23.20
89	Thr	8.55	109.38	173.53	58.48	73.17
90	Arg	-	-	175.91	54.58	33.39
91	Cys	9.12	123.35	173.15	54.58	30.78
92	Val	8.24	119.76	174.91	60.63	34.01

93	Ala	9.52	134.14	175.79	50.00	19.79
94	Arg	8.48	121.00	174.35	54.08	34.24
95	Gly	-	-	171.41	44.82	-
96	Thr	9.68	115.74	174.94	61.40	71.55
97	His	8.53	123.56	174.35	53.59	31.47
98	Ser	8.43	122.87	173.89	59.19	65.37
99	Ile	8.41	125.66	175.03	59.80	40.59
100	Glu	8.53	125.98	175.08	58.14	29.53
101	Phe	7.82	125.57	174.38	56.73	42.21
102	Met	8.62	123.91	175.85	56.31	29.46
103	Gly	8.41	103.47	174.42	45.25	-
104	Val	8.03	124.48	174.54	60.09	31.99
105	Pro					
106	Pro	-	-	177.66	62.59	32.13
107	Thr	6.60	110.14	177.01	61.38	72.52
108	Gly	8.73	112.17	173.85	45.52	
109	Arg	8.01	120.74	174.70	55.26	30.39
110	Pro	-	-	176.38	61.62	32.53
111	Phe	7.96	115.45	174.33	55.67	42.48
112	Glu	9.10	120.56	175.12	55.64	32.91
113	Met	9.26	123.62	174.43	54.40	36.13
114	Thr	9.55	124.38	172.59	64.44	69.14
115	Met	8.41	125.60	174.37	54.82	37.37
116	Leu	8.25	124.77	174.31	53.55	44.50
117	Asn	8.47	115.81	174.68	50.74	-
118	Met	8.99	122.43	174.06	-	-
119	Ser					
120	Arg	-	-	175.77	54.85	32.57
121	Val	9.10	129.23	175.40	60.64	33.91
122	Arg	9.15	126.78	176.04	55.21	33.35
123	Asp	9.53	128.67	176.00	55.52	39.73
124	Gly	8.53	102.58	173.85	45.71	-
125	Arg	7.53	117.36	175.53	53.70	33.97
126	Ile	9.26	122.17	176.46	62.70	38.94
127	Val	8.55	122.20	177.35	-	-

128	Glu					
129	His					
130	Trp					
131	Thr	7.12	114.64	171.65	58.68	68.65
132	Ile	8.43	126.25	173.76	62.87	41.98
133	Ser	7.66	119.62	174.38	57.04	66.24
134	Asp	8.33	122.55	175.76	52.19	38.26
135	Asn	8.20	122.30	177.01	57.09	38.55
136	Val	8.61	119.08	178.54	67.14	31.18
137	Thr	6.90	119.05	176.34	66.64	67.12
138	Met	6.86	121.67	177.19	59.81	32.31
139	Leu	8.05	117.78	179.67	58.27	40.01
140	Ala	7.93	121.99	181.95	54.94	17.65
141	Gln	7.92	119.45	178.82	58.71	28.39
142	Leu	7.60	117.47	177.13	55.64	42.13
143	Gly	7.67	106.00	174.80	45.72	-
144	Val	7.70	120.55	175.39	62.14	32.58
145	Lys	8.18	126.50	175.78	55.82	32.82
146	Ala	8.07	126.21	177.19	52.24	19.63
147	Ser	8.08	115.92	173.48	58.14	64.47
148	Leu	7.67	129.54	182.23	56.89	42.88
